# Identification and Functional Analysis of SlitOBP11 From *Spodoptera litura*

**DOI:** 10.3389/fphys.2021.619816

**Published:** 2021-02-11

**Authors:** Jiaojiao Luo, Zan Zhang, Dongzhen Li, Jie Liu, Kun Li, Xiao Sun, Lin He

**Affiliations:** ^1^Key Laboratory of Entomology and Pest Control Engineering, College of Plant Protection, Southwest University, Chongqing, China; ^2^Academy of Agricultural Sciences, Southwest University, Chongqing, China; ^3^State Cultivation Base of Crop Stress Biology for Southern Mountainous Land of Southwest University, Southwest University, Chongqing, China; ^4^Laboratory of Forest Pathogen Integrated Biology, Research Institute of Forestry New Technology, Chinese Academy of Forestry, Beijing, China; ^5^State Key Laboratory of Crop Stress Adaptation and Improvement, School of Life Sciences, Henan University, Kaifeng, China

**Keywords:** odorant binding protein, sex pheromone, larvae olfactory, *Spodoptera litura*, SlitOBP11

## Abstract

Odorant binding proteins (OBPs) play a key role in the olfactory recognition of insects, whose functions have been extensively studied in adult insects but rarely in larvae. In this study, one OBP (*SlitOBP11*) with high expression in larval antenna but low expression in adult antenna of *Spodoptera litura* was screened by RNA-seq and verified by quantitative real-time PCR. Furthermore, the function of *SlitOBP11* was explored by analysis of the expression patterns and prokaryotic expression of proteins as well as assays of competitive binding. Competitive binding assay demonstrated that SlitOBP11 had high binding affinity to all four female sex pheromone components, but exhibited almost no binding affinity to plant volatiles except for a low affinity to Phenylacetaldehyde and Phenethyl acetate. Homology modeling and molecular docking implied that the shape of these four sex pheromones were linear, which were appropriate for the binding channel of SlitOBP11 and the amino acid residue Asn99 of SlitOBP11 might play an important role in binding. Taken together, our results indicate that SlitOBP11 may be involved in the perception of female sex pheromones by *S. litura* larvae, and OBPs in the larvae of *S. litura* play an important role in the olfactory perception process.

## Introduction

Perception of chemical signals from the environment by olfaction is essential for the life of insects, and plays important roles in their host identification, search for mates and choice of oviposition sites (Brito et al., 2016). Generally, olfaction-related proteins in insects are classified into several major classes, including odorant binding proteins (OBPs), and chemosensory proteins (CSPs) that transport hydrophobic odorants through lymph fluid (Vogt and Riddiford, 1981), odorant receptors (ORs), and pheromone receptors (PRs) that convert chemical signals into nerve electrical signals (Jones et al., 2005), ionotropic receptors (IRs) that are a variant subfamily of ionotropic glutamate receptors for detecting volatile organic compounds (Olivier et al., 2011; Rytz et al., 2013; Zhu et al., 2018), sensory neuron membrane proteins (SNMPs) which are cofactors in the sex pheromone detection system (Vogt et al., 2009), and odorant degrading enzymes (ODEs) that break down odorant molecules (Leal, 2013). It is generally believed that the first step of the chemoreception process is the binding between OBPs and odorants (Vogt, 2005; Weiming et al., 2005). The functions of OBPs have only been identified and reported in a few species of Lepidoptera larvae, such as SexiOBP13 in *Spodoptera exigua* (Jin et al., 2015), GOBPs in *Plutella xylostella* (Zhu J. et al., 2016), and GOBPs in *Manduca sexta* (Laue, 2000). However, the specific effects of most OBPs on the behavior of larvae remain elusive.

Odorant binding proteins were first discovered in the antenna of *A. ployphemus* (Vogt and Riddiford, 1981), and are specially characterized by three interlocking disulfide bonds formed by the six highly conserved cysteines (Cys; Hekmat-Scafe et al., 2002). There are three amino acids between the second and third Cys site, and always eight intervals between the fifth and sixth Cys site (Pelosi et al., 2014). All OBPs have a similar tertiary structure, which plays an important role in maintaining their functions. Based on the number of Cys, OBPs can be divided into five classes: classic OBPs with a typical six-Cys signature; dimer OBPs with two six-Cys signatures; Plus-C OBPs with two additional conserved Cys and one proline; Minus-C OBPs with four conserved Cys; and atypical OBPs with 9–10 Cys and a long *C*-terminus (Hekmat-Scafe et al., 2002; Zhou et al., 2004). According to the sequence characteristics and homology, the OBP family in Lepidopteran insects can be subdivided into pheromone binding proteins (PBPs), antennal binding proteins (ABPx; Li et al., 2016), and general odorant binding proteins (GOBPs; Krieger et al., 1996). PBPs are known to have high expression in the antenna of male and participate in the recognition of sex pheromones (Vogt and Riddiford, 1981; Maida et al., 2005; Wen et al., 2019), while GOBPs are believed to be involved in the recognition of general odorants such as plant volatiles as well as aggregation, alarm and trace pheromones. However, the functions of PBPs and GOBPs are not always specific and unique. For example, in beet armyworm (*S. exigua*), GOBP2 even has stronger binding affinity to five sex pheromones than PBP1, while PBP1, PBP2, and PBP3 have strong binding affinity to plant volatiles (Liu et al., 2015; Liu et al., 2017). It has also been reported that some OBPs specific to non-sex pheromones have certain binding affinity to sex pheromones in *Grapholita molesta* (Li et al., 2016; Chen et al., 2018), *Adelphocoris lineolatus* (Zhang et al., 2017), and other insects. Therefore, it can be speculated that although PBPs are the main OBPs for the perception of pheromones, some other common OBPs may also be involved in pheromone perception.

*Spodoptera litura* is a pest insect that threatens many crops in Asia. Due to the long-term application of chemical pesticides, the insect has evolved high resistance to many pesticides (Sang et al., 2016). Therefore, it is highly necessary to develop some green and efficient prevention methods for this insect. Some pest control methods that adopt sexual attractants to attract adult pests have been applied in production practice, but green control methods for larval pests are still not available (Yushima et al., 1973; Rao et al., 1991). Dissection of the olfactory mechanism will help to develop better pest control strategies based on larval olfaction. In this study, an OBP (designated as *SlitOBP11*) was identified by cDNA cloning, prokaryotic expression of protein, fluorescence competitive binding and homology modeling and molecular docking. *SlitOBP11* showed higher expression in larval antenna than in adult antennae and thus may play an important role in larval olfaction. The results provide some important insights into the olfactory mechanism of *S. litura* larvae.

## Materials and Methods

### Insect Rearing and Tissue Collection

The larvae of *S. litura* were kindly provided by Prof. Shuanglin Dong from Nanjing Agricultural University, Nanjing, China. The insects were reared in a plastic rectangular box in the laboratory with the temperature of 26 ± 1°C, relative humidity of 75–80% and a 14:10 h L:D photoperiod. The larvae were fed with artificial diet and the adults were fed with 10% honey solution.

Firstly, the *OBPs* with differential expression were screened using the RNA-seq data (Supplementary Table 1) and verified by quantitative real-time PCR (qPCR) with the samples of larval heads (with antennae) at different developmental stages (eggs, 1^st^, 2^nd^, 3^rd^, 4^th^, 5^th^, and 6^th^ instar) and adult tissues from 3–4 days old male and female adults, including the head (without antennae), thorax, abdomen, leg, wing, and antenna. The antennae of the sixth instar larvae (*n* = 300) were used as the sample to investigate the expression level of *SlitOBP11* in the larval antenna, and those of the adults were used as the control. All the collected tissues were frozen with liquid nitrogen and then stored at -80°C until analysis.

### RNA Isolation and cDNA Synthesis

All the samples were dissected and placed in TRIzol^TM^ Reagent (Invitrogen, Carlsbad, CA, United States) for the extraction of total RNA. After removal of the DNA from the total RNA, the first-strand cDNA was synthesized using PrimeScript^TM^ RT Kit (DRR6210A, Takara, Dalian, China) following the manufacturer’s instructions.

### Quantitative Real-Time PCR

Quantitative real-time PCR was performed on a qTOWER 2.2 real-time PCR instrument (Analytik Jena AG, Jena, Germany) with the reagent of iQTM SYBR Green Supermix (Progema, Madison, WI, United States). The reaction system consisted of 10 μL iQTM SYBR Green Supermix 7 μL sterilized ultrapure H_2_O, 1 μL of each primer(10 μmol L^–1^) and 1 μL of the sample cDNA. The reaction conditions were 2 min at 95°C, followed by 40 cycles of 95°C for 15 s and 60°C for 30 s, finally extend for 5 s at 60°C. The significance of difference in the qPCR results was analyzed using the SPSS 19.0 Independent-Sample *T* test (*P* < 0.05) with the method of 2^–ΔΔCt^ (Livak and Schmittgen, 2001). The primer was designed using the software of Primer3 Input^1^ (Table 1), and the *SlitGAPDH* (GenBank No. HQ012003.2; Figure 2) and *SlitEF* (GenBank No. DQ192234.1; Supplementary Figure 3) genes were used as the housekeeping genes. Three biological replicates with three technical replicates were performed for each sample. The melt curves and efficiency of primers see Supplementary Figures 1, 2.

**TABLE 1:** Primers used for identification, expression analysis and expression vector construction of *SlitOBP11*.

Purpose	Sequences (5′-3′)
**Fragment verification**	
F*	ATGTTCAATTCATCGGTATT
R*	TTAGATGTCAAAGCCAAAC
**Expressiion analysis(qRT-PCR)**	
SlitOBP11-F*	ATGGTGCAGAAGGAAACAGC
SlitOBP11-R*	GCACCATCACTGACCGATTC
GAPDH-F*	CGTGTTCCTGTTGCTAAC
GAPDH-R*	CTTGACCTTCTGCTTGATAG
EF-F*	ACGCTCCCGGACACAGAGAT
EF-R*	GCTCACGGGTCTGTCCGTTC
**Design of homologous recombination primer**	
F*	GCCATGGCTGATATCGGATCCATGACAGCTGAACAGAAAGCTCT
R*	TCGAGTGCGGCCGCAAGCTTTTAGATGTCAAAGCCAAACTTAG

*F: forward primer and R: reverse primer.

### cDNA Cloning and Sequencing

The total cDNA sequence of *SlitOBP11* (GenBank accession number of XM_022970952.1) was cloned and sequenced for verification. Primers (Table 1) were designed by Primer Premier 5.0 (PREMIER Biosoft International, CA, United States). After the PCR, the products were ligated into a pGEM-T vector and transformed into *Escherichia. coli* Trans5α competent cells for sequencing to verify the sequence of SlitOBP11. The positive clones were sequenced by Chengdu Qingke Zixi Biological Technology Co., Ltd. (Chengdu, Sichuan, China). The *N*-terminal signal peptide was predicted by Signal P 5.0 Server^2^ (Armenteros et al., 2019).

### Expression and Purification of *SlitOBP11* Protein

The coding region of mature protein of SlitOBP11 was amplified by PCR using the primers listed in Table 1. Homologous recombination primers were designed with the SoSoo recombinant cloning kit (Beijing Qingke Xinye Biotechnology Co., Ltd., Beijing, China, Table 1). With restriction sites *Bam*HI and *Hin*dIII, the mature protein was ligated into a pET32a(+) vector (Novagen, Madison, WI, United States). Then, the vector was transformed into the Escherichia coli Trans5α competent cells. The monoclonal colonies were selected to confirm the presence of the insertion by sequencing. The positive vector was then transformed into BL21 (DE3) *E. coli* cells. IPTG at 0.1 mM was used to induce the expression of the protein with the cell being cultured at 24°C and 180 rpm in a table concentrator for 18 h. The expressed protein was purified by Ni-NTA Sefinose^TM^ Resin Kit following the manufacturer’s instructions. In order to qualitatively determine whether the protein was the target protein, western blotting was performed as follows. The purified protein was subjected to SDS-PAGE electrophoresis. The SDS-PAGE gel was transferred to a polyvinylidene difluoride membrane (PVDF), and then blocked with 5% skim milk in TBST for 1 h. Then, the PVDF membrane after overnight incubation was washed with TBST for three times, followed by the addition of diluted Goat Anti-Rabbit IgG (H + L; 1:20000) secondary antibody and incubation for 1 h. ECL Chemiluminescence Substrate Kit (Bio-Rad Company) was used for the development of western blots according to the instructions (Figure 3). Enterokinase was used to cleave the His-tag, and the OBP was filtered out by the Ni-NTA Sefinose^TM^ Resin Kit. Then, the purified protein solution was dialyzed to remove the ions, and determined for concentration and stored at -80°C.

### Binding Affinity of *SlitOBP11*

The analysis of affinity characteristics was performed with the method reported by Ban et al. (2002). Binding experiment was performed on a F97pro Fluorescence Spectrophotometer (Shanghai Lengguang Technology Co., Shanghai, China). A total of 48 odorant compounds were used to test the function of SlitOBP11, including four female sex pheromone components and most of host plant (Cabbage) volatiles of *S. litura* (Table 2). All the odorant compounds except for the sex pheromone components and the fluorescent probe 1-N-Phenyl-naphthylamine (1-NPN) were purchased from Sigma-Aldrich (purity ≥ 95%), and the four female sex pheromone components (purity ≥ 95%) were synthesized by Jiangsu Ninglu Technology Co., Ltd. (Changzhou, China). All the odorants were dissolved in methanol as 100 mM stock solution.

**TABLE 2:** Binding affinities of SlitOBP11 for plant volatile compounds and female sex pheromones of *S. litura*.

Odorant	IC_50_ (μmol L^–1^)	K*_*i*_* (μmol L^–1^)^∗^
**Sex pheromone component**		
*(Z, E)*-9,11-tetradecadienyl acetate	15.56 ± 1.407	13.88 ± 1.4412
*(Z, E)*-9,12-tetradecadienyl acetate	16.19 ± 0.32	14.40 ± 0.3173
*(Z)*-9-tetradecenyl acetate	11.21 ± 0.55	9.97 ± 0.4923
*(E)*-11-tetradecenly acetate	15.24 ± 0.54	13.53 ± 0.4789
**Aromatic compounds**		
Benzaldehyde	>50	—
Phenylacetaldehyde	43.36 ± 10.07	35.83 ± 9.7553
Phenethyl alcohol	>50	—
Benzyl alcohol	>50	—
Benzyl acetate	>50	—
Phenethyl acetate	48.59 ± 0.52	43.06 ± 0.4882
Methyl anthranilate	>50	—
Ethylbenzene	>50	—
**Methyl ketone compounds**		
3-hexanone	>50	—
2-hexanone	>50	—
**Terpenoids**		
(+)-Carvone	>50	—
Geranylacetone	>50	—
(±)-Linalool	>50	—
Beta-caryophyllene	>50	—
Farnesene	>50	—
*Trans*-nerolidol	>50	—
Alpha-phellandrene	>50	—
*(R)*-(+)-limonene	>50	—
Myrcene	>50	—
Geranyl acetate	>50	—
**Heterocyclic compounds**		
Eucalyptol	>50	—
Indole	>50	—
**Lipid compound**		
Tridecane	>50	—
Tetradecane	>50	—
*Cis*-3-hexenyl acetate	>50	—
*Alpha*- ionol	>50	—
*Beta*-ionone	>50	—
Methyl salicylate	>50	—
*N*-octadecane	>50	—
Octyl aldehyde	>50	—
Dodecane	>50	—
Decanal	>50	—
Acid compounds		
Acetic acid	>50	—
Propanoic acid	>50	—
Butyric acid	>50	—
Isobutyric acid	>50	—
Pentanoic acid	>50	—
2-methylbutyric acid	>50	—
3-methylbutyric acid	>50	—
Hexanoic acid	>50	—
3-methylpentanoic acid	>50	—
4-methylpentanoic acid	>50	—
Heptanoic acid	>50	—
Crotonic acid	>50	—

*For values of IC_50_ higher than 50 μmol L^–1^, the corresponding K*_*i*_* values were not calculated and are indicated with “-”.

A 2 μM solution of protein in 30 mM *tris*-HCl buffers with pH 7.4 was confected and then titrated with aliquots of 1 mM 1-NPN to final concentrations of 2–24 μM to determine the affinity of 1-NPN to SlitOBP11. To further measure the affinities of other ligands to SlitOBP11 by competitive binding assays, SlitOBP11 and 1-NPN at 2 μM were titrated with aliquots of 1 mM competitor to final concentrations of 0–10 μM for female sex pheromone components and 0–20 μM for other odorant compounds. The excitation wavelength was 337 nm, and the scanning wavelength range was 360–550 nm. The excitation slit was 20 nm, and the emission slit was 20 nm. The scanning at a medium speed with sensitivity was set to 1 and the temperature was 4°C.

The binding data detected for the affinity assays between 1-NPN and SlitOBP11 were calculated by Scatchard plots for the dissociation constants of K_1__–NPN_ on the assumption that the stoichiometry of protein: ligand was 1:1 at saturation. The value of IC_50_ (concentrations of ligands halving the initial fluorescence value of 1-NPN) was used to calculate the dissociation constant (K*_*i*_*) of competitors to SlitOBP11 with the equation of K*_*i*_* = [IC_50_]/(1-[NPN]/K_1__–NPN_), where [1-NPN] is the free concentration of 1-NPN.

### Homology Modeling and Molecular Docking

The structure of SlitOBP11 was predicted by homology modeling. The SWISS.MODEL^3^ was used to find template protein for homology modeling. Finally, the OBP56a (PDB ID: 5DIC) of *Phormia regina* was selected as homology model (similarity 34.23%). Then, the Molecular Operating Environment (MOE, version 2012.10) was used for homology modeling. The ‘maximum number of mainchain models’ was set to 50 and ‘sidechain samples at temperature 300 K’ was set to 5 in the modeling procedure, and ‘intermediates’ and the ‘final model’ were set to “fine”, and AMBER99 was selected as the force field in the model refinement section, while other parameters were set to default. The model was subjected to sufficient energy minimization and sufficient stereochemical refinement according the electrostatic solvation energy, which was calculated using the Generalized Born/Volume Integral methodology. The best SlitOBP11 model with the lowest electrostatic solvation energy and optimal geometric properties was selected for follow-up analysis.

After the tertiary structures was determined, (Z,E)-9,11-Tetradecadienyl acetate (PubChem CID: 6441057), (Z,E)-9,12-Tetradecadienyl acetate (PubChem CID: 5365642), (E)-11-Tetradecenyl acetate (PubChem CID: 5367650), (Z)-9-Tetradecenyl acetate (PubChem CID: 5364714) volatiles were docked into the cavity of SlitOBP11, and this was built using the Surflex-Dock suite embedded in Sybyl-X (version 2.0). In this process, Surflex-Dock was selected as the docking mode and a Multi-Channel Surface was set as the protomol generation mode, ‘bloat’ was set to 2 Å, ‘additional starting conformations per molecule’ was increased to 10, ‘density of search’ was set to 6 and ‘consider ring flexibility’ was checked. Finally, ‘minimum RMSD (root-mean-square deviation) between final poses’ was set to 0.5 Å to explore additional docking poses and to achieve higher accuracy and other parameters were set to default.

## Results

### Gene Cloning and Sequence Analysis of *SlitOBP11*

**FIGURE 1:**
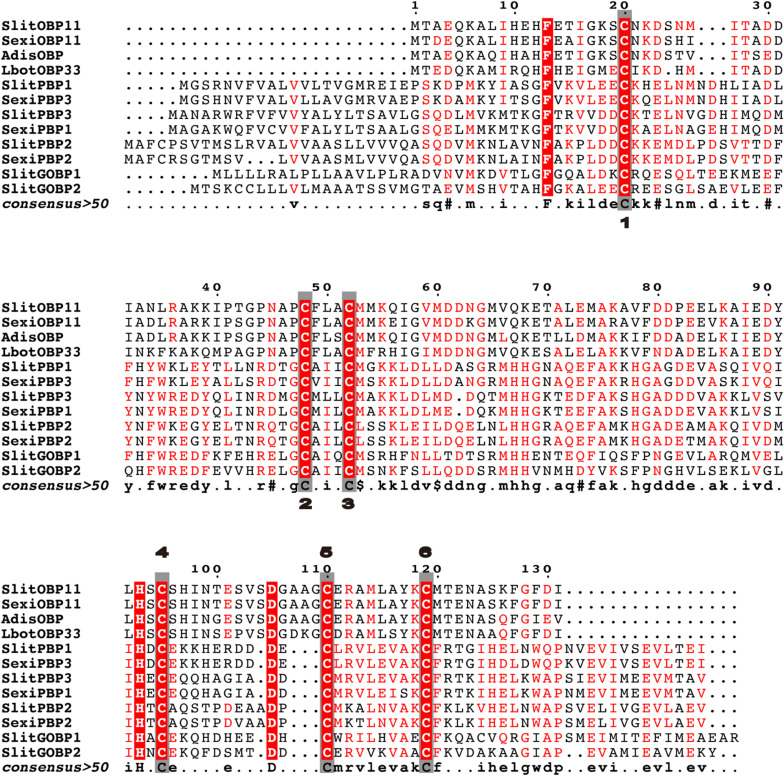
Alignment of SlitOBP11 with SexiOBP11, SexiPBP1, SexiPBP2, SexiPBP3 from *Spodoptera exigua*, AdisOBP from *Athetis dissimilis*, and LbotOBP33 from *Lobesia botrana*. Conserved cysteines are highlighted in the gray box and marked with numbers. The GenBank accession number is XM_022970952.1 for SlitOBP11, AGP03457.1 for SexiOBP11, QCF41969.1 for AdisOBP, AXF48730.1 for LbotOBP33, AKI87957.1 for SlitPBP1, AKI87958.1 for SlitPBP2, AKI87959.1 for SlitPBP3, AKI87960.1 for SlitGOBP1, AKI87961.1 for SlitGOBP2, AAU95536.1 for SexiPBP1, AAU95537.1 for SexiPBP2, and ACY78413.1 for SexiPBP3. The sequences were aligned by Multalin and figure was created by ESPript.

The sequence of SlitOBP11 was downloaded from NCBI with the GenBank accession number of XM_022970952.1 and verified by gene cloning. There was an open reading frame of 465 bp encoding 155 amino acids in SlitOBP11. According to the Signal P 5.0 server (see text footnote 2), 23 amino acids were predicted as the signal peptide at the hydrophobic *N*-terminus. The protein sequence of *SlitOBP11* with all structural characteristics of an OBP gene (Vogt and Riddiford, 1981; Zhou et al., 2008) had six conserved Cys (Figure 1), and shared high identity with SexiOBP11 (90.26%) and SexiPBP3 (87.20%) from *S. exigua*, AdisOBP from *Athetis dissimilis* (83.21%), LbotOBP33 from *Lobesia botrana* (71.97%; Rojas et al., 2018; Figure 1), and low identity with SlitPBP1(19.69%), SlitPBP2(44.97%), SlitPBP3(48.17%), SlitGOBP1(30.43%), SlitGOBP2(28.57%) from *S. litura* and SexiPBP1(48.78%), SexiPBP2(45.18%) from *S. exigua*. The sequences were aligned by Multalin (available at http://bioinfo.genopole-toulouse.prd.fr/multalin/multalin.html) and figure was created by ESPript (Gouet et al., 1999; Figure 1).

**FIGURE 2:**
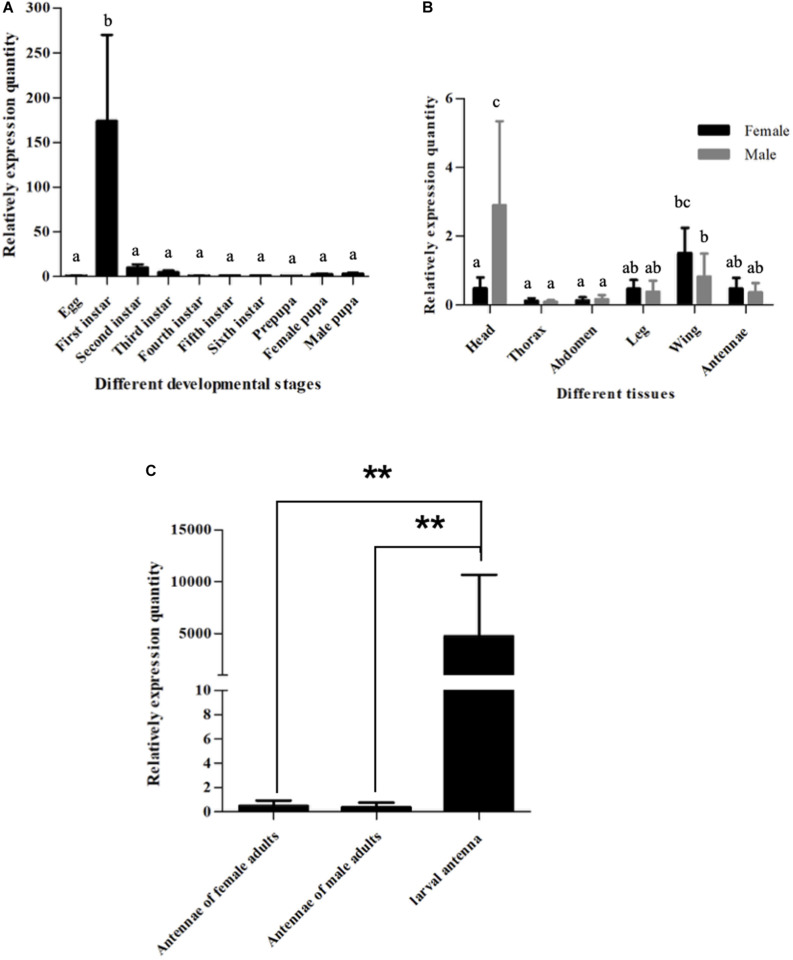
Expression patterns of *SlitOBP11*. **(A)** Expression patterns of *SlitOBP11* at different developmental stages of *S. litura*. **(B)** Expression patterns of *SlitOBP11* in different tissues of *S. litura* adults [head (without antenna)]. **(C)** Expression of *SlitOBP11* in the antenna of larvae and adults. ‘**’ Means *T* test *p* < 0.01.

**FIGURE 3:**
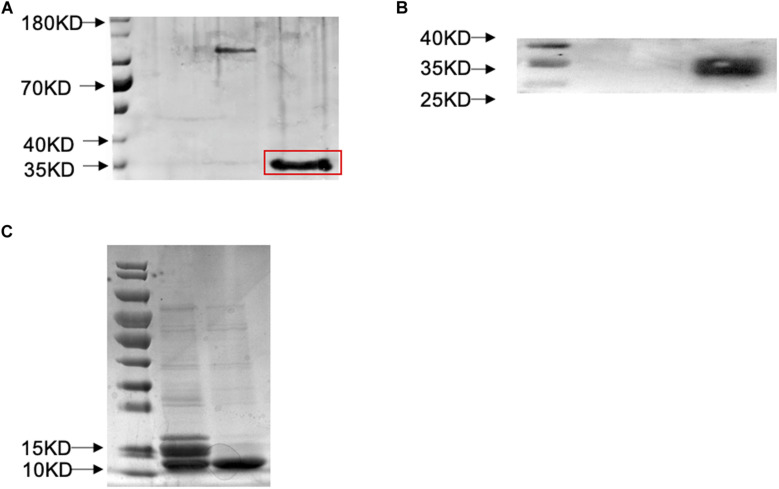
Prokaryotic expression and western blot verification of SlitOBP11. **(A)** SDS-PAGE was used to detect the expression of SlitOBP11 with His-tag after induction with IPTG and purification by Ni-NTA column [The band is highlighted by a red box, and the tags were from pET32a(+), which has a label of about 20 KD.]. **(B)** Western blot was used to confirm the results of purification; well 2 shows the SlitOBP11 and well one is blank. **(C)** SDS-PAGE was used to detect the SlitOBP11 after cleavage of the His-tag (The first electrophoresis Lane, enterokinase was used to cleave the His-tag) and purification by Ni-NTA column (The second electrophoresis Lane).

### Expression Pattern of *SlitOBP11*

To further verify the expression pattern *SlitOBP11*, we determined its expression in the heads (with antennae) of larvae and various tissues of adults. As a result, *SlitOBP11* was highly expressed in the heads with antennae in the first instar larvae, and lowly expressed in the eggs and heads with antennae of the second to sixth instar larvae as well as in male prepupa and female pupa (Figure 2A). For different tissues of adults, *SlitOBP11* showed similar expression patterns between female and male in the antennae, heads, thorax, abdomen, wings, and legs. The expression was the highest in the heads, followed by the wings, antennae, legs, abdomen, and thorax (Figure 2B). Because of the difficulty in obtaining the antennae of the first to fifth instar larvae, only the sixth instar larval antenna could be dissected for RNA extraction, and the analysis of the expression level showed that *SlitOBP11* had higher expression in larval antenna (Figure 2C). We also used the *SlitEF* as housekeeping gene to investigate the expression pattern of *SlitOBP11*. And though there was a difference between two housekeeping genes (*SlitEF*, *SlitGAPDH*), the analysis results of *SlitEF* as housekeeping gene displayed the same expression pattern of *SlitOBP11* as the *SlitGAPDH.*

### Affinity Analysis of SlitOBP11

To further clarify the binding characteristics of SlitOBP11, a prokaryotic expression system was used to obtain the recombinant protein. Then, SlitOBP11 was purified in supernatant through a His-tag affinity column and the His-tag was subsequently removed with enterokinase (Figure 3).

**FIGURE 4:**
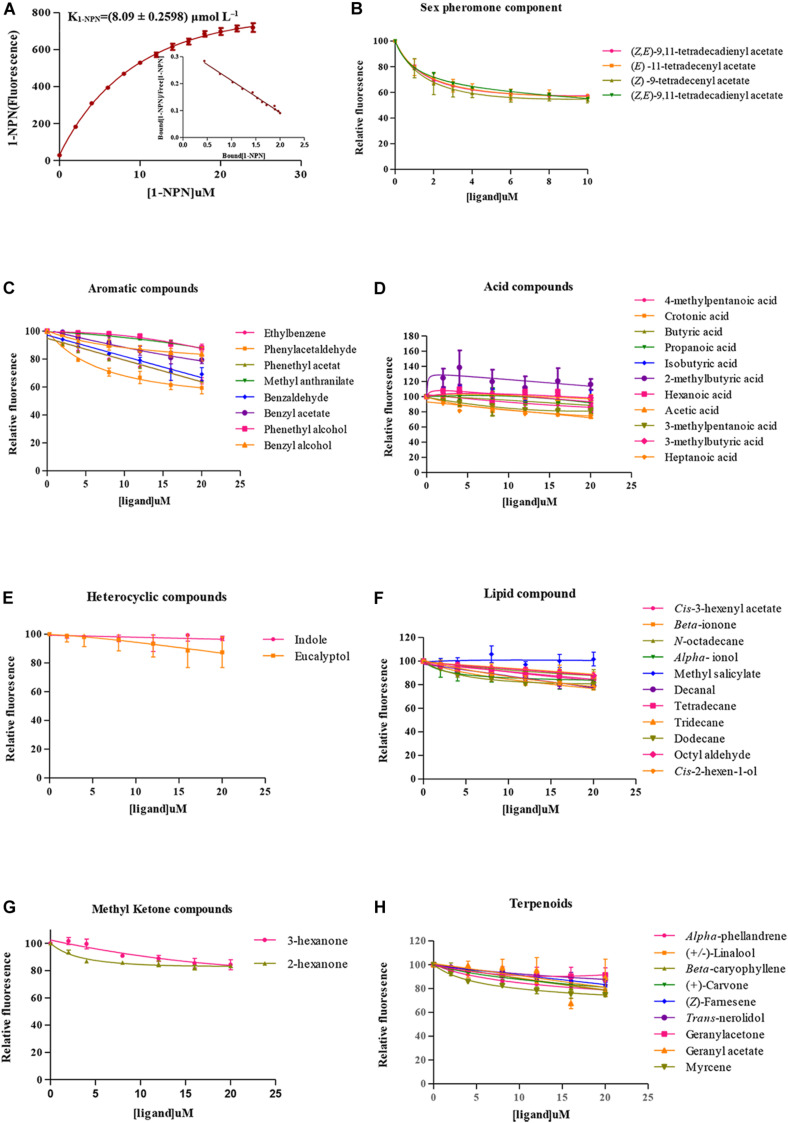
Fluorescence competitive binding assay. **(A)** Binding curve of 1-NPN to SlitOBP11 and Scatchard plot. **(B)** Binding curve of SlitOBP11 with sex pheromones of female. **(C–H)** Binding curve of SlitOBP11 with plant volatile compounds.

The probe of 1-NPN was used to investigate the binding characteristics of SlitOBP11. The binding curve between 1-NPN and SlitOBP11 was obtained for the measurement of the dissociation constant. As a result, the dissociation constant was 8.09 ± 0.2598 μmol L^–1^ (Figure 4A). With increasing content of 1-NPN, a saturation and a linear Scatchard plot were observed, indicating a single binding site and no allosteric effect.

A total of 48 odorants were tested in the competitive binding assays. As shown in Figure 4, only six odorants were screened to have binding affinity to SlitOBP11 based on the cut off values of K*_*i*_* < 50 μmol L^–1^. Among these odorants, four were female sex pheromones with the binding affinity of K*_*i*_* = 13.88 ± 1.4412 μmol L^–1^ for Z9,E11–14:Ac, K*_*i*_* = 14.40 ± 0.3173 μmol L^–1^ for Z9,E12–14:Ac, K*_*i*_* = 9.97 ± 0.4923 μmol L^–1^ for Z9–14:Ac, and K*_*i*_* = 13.53 ± 0.4789 μmol L^–1^ for E11–14:Ac. The remaining two odorants with certain binding affinity for SlitOBP11 were Phenylacetaldehyde (K*_*i*_* = 35.83 ± 9.7553 μmol L^–1^) and Phenethyl acetate (K*_*i*_* = 43.06 ± 0.4882 μmol L^–1^).

### Homology Modeling and Molecular Docking of SlitOBP11

**FIGURE 5:**
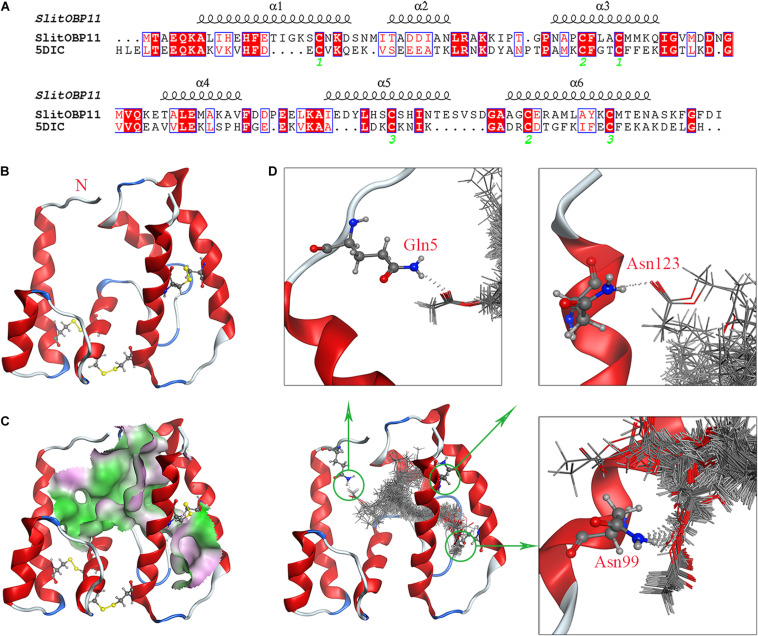
Homology modeling and molecular docking of SlitOBP11. **(A)** Sequence alignment of SlitOBP11 and the template 5DIC. The secondary elements of SlitOBP11 are shown above the sequences. The green number showed the formation of disulfide bond. **(B)** Predicted 3D model of SlitOBP11. There are six Cys residues and three disulfide bridges between Cys residues Cys20-Cys52 and Cys48-Cys110 and Cys95-Cys119, which form six α-helix. **(C)** An open cavity of SlitOBP11 at the center of the protein core. The green areas express hydrophobicity and red areas express hydrophilia of binding cavity. **(D)** Predicted formation of hydrogen bond between cavity and ligands. Three hydrophilic residues Gln5, Asn99, and Asn123 form the polar surface of the cavity. The red atom is oxygen atom. The blue atom is nitrogen-atom. The gray molecules in the cavity different ligands.

The research results of SWISS.MODEL showed OBP56a (PDB ID: 5DIC) of *Phormia regina* had the highest sequence similarity with SlitOBP11, which was the only alternative template with a sequence similarity above 34%. Considering the highly conserved Cys residues, SlitOBP11 and 5DIC shared six Cys residues at conserved sites (Figure 5A). Although there were three gaps in alignment analysis, subsequent analysis showed that these gaps were located far from the structural center, which was unexpected to form the binding cavity of SlitOBP11. Therefore, 5DIC was selected as the template for homology modeling. Depended on the stereo-chemical optimization and energy minimization, the first-rank model with the minimum energy model was inspected using the stereo-chemical quality evaluation tool in MOE-Protein Geometry. A pairwise RMSD of alpha C between the template 5DIC and SlitOBP11 was 1.86 Å (Supplementary Figure 4B). As shown in Supplementary Figure 4A, most residues were located in the allowed region in a Ramachandran map, along with other stereochemical indices (including bond lengths, bond angles, and dihedrals), indicating that its overall stereochemical quality was generally reliable and acceptable.

SlitOBP11 had six Cys residues and three disulfide bridges between Cys residues Cys20-Cys52 and Cys48-Cys110 and Cys95-Cys119. Compared with template 5DIC, a longer α-helix 5 can be observed (Figures 5A,B). Four female sex pheromone components were docked into the cavity of SlitOBP11 to investigate the recognition mechanism. At the center of the protein core, an open cavity was shown, which liked a channel. And the cavity walls are principally formed of hydrophobic residues (Figure 5C), which indicated the hydrophobic interaction and van der Waals interaction force maybe the primary forces between ligands and cavity. Additionally, three hydrophilic residues are also part of the cavity wall: Gln5, Asn99, and Asn123, which forms the polar surface of the cavity. The docking results with a variety of possible ligands orientation in cavity indicated that these three residues provided potential sites for hydrogen bond. Among them, the biggest probability mainly existed in Asn99, which implied Asn99 may be the key amino acid in ligand-binding (Figure 5D).

## Discussion

A variety of crops suffer from severe damage caused by Lepidoptera insects mostly at the larval stage. The host of Lepidoptera larvae is selected by the previous generation of female when laying eggs (Petit et al., 2015). It has been reported that Lepidoptera larvae can locate the source of odorants and accordingly change their host plants (Poivet et al., 2012). However, their ability to select host plants has always been neglected in the studies of larval feeding behavior (Jaenike, 1978; Carrasco et al., 2015). The young larvae of Lepidoptera are characterized by the habit of aggregated feeding (Denno and Benrey, 1997; Jin et al., 2016). For Lepidoptera insects that lay eggs in the form of egg blocks, it is generally believed that after hatching, the larvae naturally gather near the spawning position for convenient feeding and resistance against natural enemies and plant immunity together (Clark and Faeth, 1997; Despland, 2019). Recent research has revealed that the sex pheromone components left by female spawning are key factors that lead to the aggregation of the hatched offspring (Poivet et al., 2012; Jin et al., 2015; Zhu G. H. et al., 2016; Zielonka et al., 2016). The aggregation behavior improves the survival rate of young larvae and enhances their ability to resist against harsh environments (Morimoto et al., 2018). These special characteristics of odor-induced larval aggregation and feeding provide a new research perspective to use sex pheromones as an effective tool for the biological control of Lepidoptera larvae (Poivet et al., 2012).

Compared with that of adults, the olfactory mechanism of larvae has been much less studied. It is generally believed that larvae have the same molecular mechanism of olfaction as adults, both of which involve OBPs and ORs as the key proteins (Zhu J. et al., 2016). However, there may be differences between larvae and adults in the functional genes that specifically target the same odorant molecules. For example, the OBPs highly expressed in *Drosophila melanogaster* larvae are not consistent with those in the adults, and the larvae and adults may rely on different OBPs to perceive the smell of fruit (Dweck et al., 2018). As for the molecular mechanism of sex pheromone perception, some researchers believe that the larvae may use part (PBPs and PRs) of the olfactory system of the adults for the perception of sex pheromones. The larvae of *Spodoptera littoralis* could sense the main components (Z9, E11–14: Ac) of sex pheromones and be attracted by them. It has been found that adult PBPs are expressed in the antenna of larvae, but no PRs are expressed. Besides, it was reported that the larvae of *S. littoralis* may use PBPs for Z9,E11–14:Ac transport, and the receptor of Z9,E11–14:Ac is not the prs gene (Poivet et al., 2012), but possibly an unknown OR gene. Some researchers proposed that larvae might have a totally different olfactory perception system that involves other OBPs and ORs instead of PBPs and PRs for the perception of sex pheromones. In beet armyworm (*S. exigua*), it was also observed that the main components of sex pheromones can attract larvae, and SexiOBP13 is specifically expressed in the larvae with a specific binding affinity to the main components of sex pheromones (Jin et al., 2015). In the heads of diamondback moth (*P. xylostella*), the expression of five genes with important functions in adults, including GOBP1, GOBP2, PBP1, PBP2, and PBP3, was determined in the first to fourth instar larvae. As a result, only the expression of PBP1 was detected in the third instar larvae, while that of GOBP1 and GOBP2 was detected in all the four instar larvae, indicating that GOBP may substitute PBP to participate in the perception of sex pheromones in young larvae (Zhu J. et al., 2016). At present, limited research has been focused on the olfactory sense and related genes of young Lepidoptera larvae. The mechanism of sex pheromone perception of young Lepidoptera larvae is still unclear and remains to be dissected in future studies.

The binding mechanism of OBPs with ligands were always complex, especially the specificity. Similar with other studies, the predicted cavity of SlitOBP11 mainly was hydrophobic, which was the typical characteristic of OBPs structure (Zhou et al., 2009; Lagarde et al., 2011; Yang et al., 2017). Although the hydrophobic wall was helpful to bind with odor that mainly were hydrophobic molecules in environment, the hydrophobic interaction and van der Waals interaction, that mainly depended on enough touch between cavity and ligands (Leite et al., 2009; Li et al., 2015), commonly could not provide desirable binding selectivity. Hydrogen-bond seemed a preferable manner for the ligands with polar atoms (Kruse et al., 2003; Thode et al., 2008; Zhuang et al., 2014). Along with the conformational flexibility of OBPs (Davrazou et al., 2011; Spinelli et al., 2012), they lead the relative broad binding capacity. In our docking models, the shape of these four sex pheromones were linear, which were appropriate for the binding channel. As the binding character of OBPs with linear ligands, the long-chain and flexibility of sex pheromones maybe lead well hydrophobic interaction (Mao et al., 2010; Zheng et al., 2016). Meanwhile, all the four sex pheromones had oxygen atoms, and the possible hydrogen-bonds were formed in cavity. Especially, the amino acid residue Asn99, which was predicted located in the deep of cavity and provided more possibilities for hydrogen-bond formation, might play an important role in binding. However, it was unclear whether the specific functional group of sex pheromones were the critical factor for specific binding. In *Culex quinquefasciatus*, CquiOBP1 recognizes the length of the lipid chain that fits its hydrophobic tunnel instead of specific functional group of MOP [(5R,6S)-6-acetoxy-5-hexadecanolide] (Mao et al., 2010). So, more research are needed to understand the binding mechanism of SlitOBP11 with these four sex pheromones.

In this study, we screened one OBP (*SlitOBP11*) which has higher expression in larval antenna than in adult antenna. The expression pattern of *SlitOBP11* suggested that it has certain functions in the antennae of larvae, and more specifically, it may be involved in the olfactory process of larvae. Our competitive binding assays revealed that the protein has high binding affinity to all four female sex pheromone components, indicating that SlitOBP11 may be involved in the perception of sex pheromone components by larvae. And homology modeling and molecular docking revealed that the shape of these four sex pheromones were appropriate for the binding channel of SlitOBP11. Our results imply that there may be a new set of OBPs for the larvae to perceive sex pheromone components.

In general, in this study, the full-length cDNA of *SlitOBP11* was cloned and *SlitOBP11* was found to have higher expression in larval antenna than in adult antenna. Further analysis showed that the expression was particularly high in the head of the first instar larvae. The competitive binding indicated that SlitOBP11 may be involved in the perception of female sex pheromones by *S. litura* larvae. Homology modeling and molecular docking revealed that the shape of these four sex pheromones were appropriate for the binding channel of SlitOBP11 and the amino acid residue Asn99 of SlitOBP11 might play an important role in binding. Our study suggests that there may be a new set of OBPs for the larvae to perceive sex pheromone components.

## Data Availability Statement

The datasets presented in this study can be found in online repositories. The names of the repository/repositories and accession number(s) can be found in the article/Supplementary Material.

## Author Contributions

JLuo, XS, and ZZ wrote the initial manuscript, analyzed the data, and prepared the figures. ZZ provided conception and design of research. DLi provided the homology modeling and molecular docking. JLiu provided experimental method support. JLuo and ZZ performed the experiments. ZZ and LH wrote, edited, and reviewed the manuscript. All authors accepted the final version of the manuscript.

## Conflict of Interest

The authors declare that the research was conducted in the absence of any commercial or financial relationships that could be construed as a potential conflict of interest.

## Footnotes

1 http://frodo.wi.mit.edu/

2 http://www.cbs.dtu.dk/services/SignalP/

3 https://swissmodel.expasy.org/
