# Supplementary material for: Identification and Functional Analysis of SlitOBP11 From Spodoptera litura
Source: Front Physiol. 2021 Feb 11;12:619816. doi: 10.3389/fphys.2021.619816 (PMC7904875; doi:10.3389/fphys.2021.619816)
Supplement: Supplementary file 1 [file Table_1.docx]

**Supplements 1: RNA-Seq data assemble and difference expression gene analysis**

Download and analysis of RNA-Seq data

The RNA-seq data were download from NCBI with the SRA No. SRX2554482(female antennae), SRX2554481(male antennae), SRX2554474(antennae of 6^th^ instar larvae) and SRX2554458(antennae of 6^th^ instar larvae). The Hisat2 was used to map all the reads to the genome of S. litura, following with the StringTie to assemble the mapped reads into transcripts and evaluate the expression of each gene in the transcriptome. The default parameters were used for Hisat2(Kim et al., 2019) and StringTie(Pertea et al., 2016). On the basis of StringTie assembly,the read counts of the transcriptome were obtained from the results of StringTie, and then, the DESeq(Anders and Huber, 2012) was used to analyze the differentially expressed genes between the larvae and adult in antennae. The two RNA-Seq data (SRX2554474 and SRX2554458) of larvae antennae was used to compare with female antennae (SRX2554482) and male antennae (SRX2554458). Furthermore, the higher expressed odorant binding proteins(OBP) in larvae antennae than adult antennae were selected and used to study the mechanism of the larval olfactory perception(Table S1, cutoff: |log2FoldChange| > 2 and Padj < 0.01).

**Table S1.** higher OBPs in larvae antennae than in adult antennae,and the *gene10328* was *SlitOBP11*

| Transcriptome ids | Log2FoldChange(m:l)* | Pvalue(m:l) | Padj(m:l) | Log2FoldChange(l:f)+ | Pvalue(l:f) | Padj(l:f) | RNA ids in genome | Description |
| --- | --- | --- | --- | --- | --- | --- | --- | --- |
| gene10329 | -19.09838933 | 1.82E-20 | 5.45E-19 | 14.70587491 | 7.59E-81 | 1.89E-78 | XM_022970953.1 | Odorant-binding protein |
| gene10347 | -5.60734574 | 1.12E-05 | 8.20E-05 | 3.781881843 | 0.000507639 | 0.002687981 | XM_022971005.1 | Odorant-binding protein |
| gene10328 | -11.20391 | 3.04E-178 | 5.67E-175 | 10.9909 | 2.21E-189 | 3.16E-186 | XM_022970952.1 | odorant-binding protein 11 |
| gene8234 | -6.383866569 | 0.000280262 | 0.001552944 | 7.018287908 | 2.21E-05 | 0.00015406 | XM_022968052.1 | Odorant-binding protein 1 |
| gene10330 | -10.4868127 | 0.000173903 | 0.00101216 | 10.5356443 | 0.000114008 | 0.000693753 | XM_022971256.1 | Odorant-binding protein |

* m:l means that the gene expression in male antennae compare with that in larvae antennae.

^+^ l:f means that the gene expression in larvae antennae compare with that in female antennae.

The details of Log2FoldChange, pvalue and padj see the manual of DESeq (http://bioconductor.org/packages/release/bioc/html/DESeq.html).

**References:**

Anders, S., and Huber, W. (2012). Differential expression of RNA-Seq data at the gene level–the DESeq package. Heidelberg, Germany: European Molecular Biology Laboratory (EMBL) 10, f1000research.

Kim, D., Paggi, J.M., Park, C., Bennett, C., and Salzberg, S.L. (2019). Graph-based genome alignment and genotyping with HISAT2 and HISAT-genotype. Nature biotechnology 37, 907-915.

Pertea, M., Kim, D., Pertea, G.M., Leek, J.T., and Salzberg, S.L. (2016). Transcript-level expression analysis of RNA-seq experiments with HISAT, StringTie and Ballgown. Nature protocols 11, 1650.
