# Supplementary material for: Identification and Functional Analysis of SlitOBP11 From Spodoptera litura
Source: Front Physiol. 2021 Feb 11;12:619816. doi: 10.3389/fphys.2021.619816 (PMC7904875; doi:10.3389/fphys.2021.619816)

**Supplements 2: Melt curves and efficiency of primers**

**Figure S1.** The melt curves of primer, the template for detection was from 6^th^ instar of whole-body larvae. (A)melt curves of *SlitEF* primers. (B) melt curves of *SlitGAPDH* primers. (C)melt curves of *SlitOBP11* primers. The yellow was the melt curves with the template quality of 1μg, light red was 0.3333μg, light green was 0.1111μg, red was 0.0370μg. green was 0.0123μg and blue was 0.0041μg.


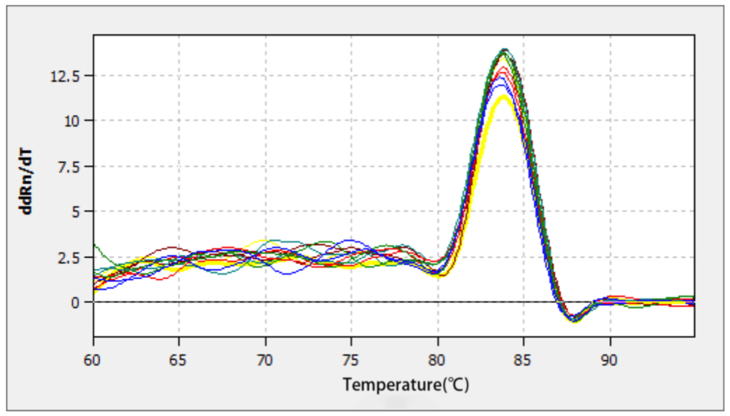


**A**


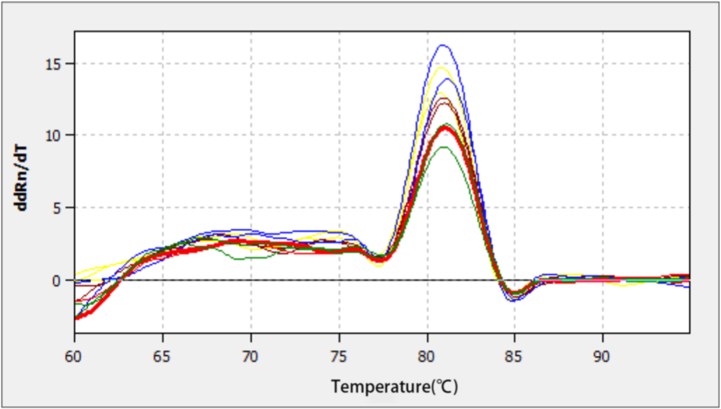


**B**


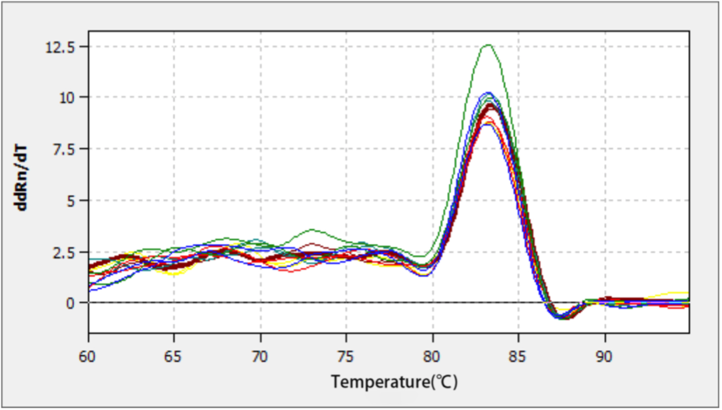


**C**

**Figure S2.** The standard curve and efficiency for each gene primers. (A) standard curve of of *SlitEF* primers with the primers efficiency of 1.01(R^2^:0.99348). (B) standard curve of of *SlitGAPDH* primers with the primers efficiency of 0.94 (R^2^: 0.99506). (C) standard curve of of *SlitOBP11* primers with the primers efficiency of 1.08 (R^2^: 0.99178).


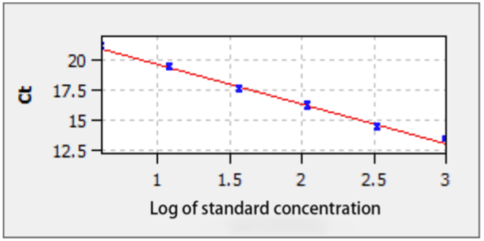


**A**


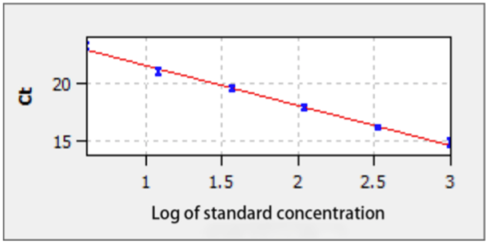


**B**
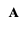


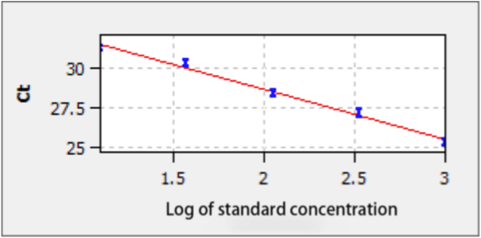


**C**
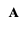

Supplement: Supplementary file 2 [file Table_2.docx]
