# Supplementary material for: Identification and Functional Analysis of SlitOBP11 From Spodoptera litura
Source: Front Physiol. 2021 Feb 11;12:619816. doi: 10.3389/fphys.2021.619816 (PMC7904875; doi:10.3389/fphys.2021.619816)
Supplement: Supplementary file 3 [file Table_3.docx]

**Supplements 3: Quantitative Real-Time PCR for *SlitOBP11* in different instar of *S. litura* larvae and different tissues of *S. litura* adults with *SlitEF* as housekeeping gene.**

**Figure S3.** Expression patterns of *SlitOBP11*. (A) Expression patterns of *SlitOBP11* at different developmental stages of *S. litura*. (B) Expression patterns of *SlitOBP11* in different tissues of *S. litura* adults. (C) Expression of *SlitOBP11* in the antenna of larvae and adults.
