# Supplementary material for: Identification and Functional Analysis of SlitOBP11 From Spodoptera litura
Source: Front Physiol. 2021 Feb 11;12:619816. doi: 10.3389/fphys.2021.619816 (PMC7904875; doi:10.3389/fphys.2021.619816)
Supplement: Supplementary file 4 [file Table_4.docx]

**Supplements 4: Evaluation of modeling results for SlitOBP11**

**Figure S4.** (A) The Ramachandran map of the model of SlitOBP11. The scope of orange dashed line expresses the allowed region. The scope of green dashed line expresses the core region. The yellow circle means the amino acid located within the allowed region. The green circle means the amino acid located within the core region. The red cross means the amino acid that is outlier. (B) The pairwise RMSD between the template 5DIC and SlitOBP11

**
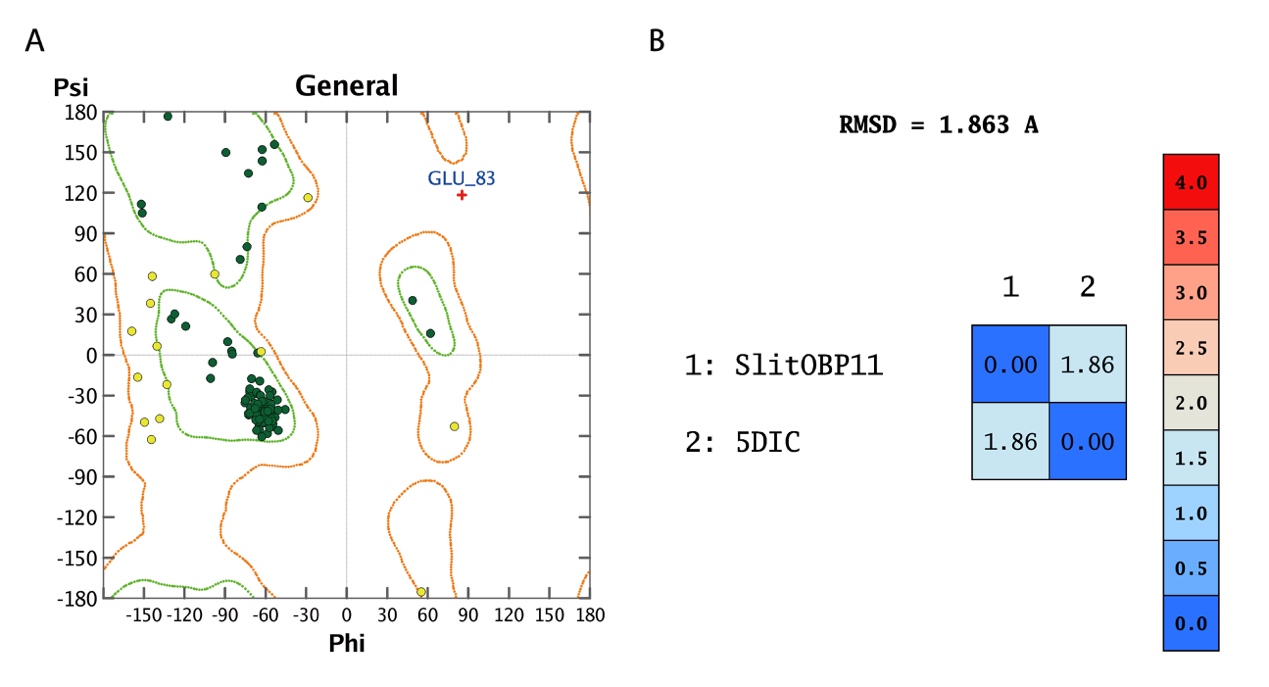
**
